# Co-expression and prognosis analyses of GLUT1–4 and RB1 in breast cancer

**DOI:** 10.1186/s12885-021-08763-y

**Published:** 2021-09-15

**Authors:** Xiaodan Zhang, Xiaocong Pang, Zhuo Zhang, Qianxin Liu, Hanxu Zhang, Qian Xiang, Yimin Cui

**Affiliations:** 1 Department of Pharmacy, Base for Clinical Trial, Peking University First Hospital, No. 8, Xishku Street, Beijing, 100034 P. R. China; 2 Institute of Clinical Pharmacology, Peking University, No.38, Xue Yuan Street, Haidian District, Beijing, 100191 China; 3 Department of Pharmacy Administration and Clinical Pharmacy, School of Pharmaceutical Sciences, Peking University Health Science Center, Beijing, 100191 P. R. China

**Keywords:** Triple-negative breast cancer, Glucose transporters, Metabolic inhibitory therapy, Individualized treatment, Metabolic plasticity

## Abstract

**Background:**

Current treatment methods for patients with triple-negative breast cancer (TNBC) are very limited, and the prognosis of TNBC is relatively poor. It has been reported that glucose transporter 1 (GLUT1) is overexpressed in breast cancer cells; however, its association with the prognosis is mostly unclear. Moreover, retinoblastoma gene 1 (RB1) might be used as a biomarker for the sensitivity of breast cancer cells to GLUT1 inhibitors, which brought us to the hypothesis that there might be a close correlation between the expression of GLUT1–4 and the expression of RB1.

**Methods:**

In this study, we systematically analyzed the co-expression of GLUT1–4 and the influence of GLUT1–4 gene expression on the prognosis of breast cancer using data mining methods. We also explored possible relationships between GLUT1–4 and RB1 expression in breast cancer tissues. We used public databases such as ONCOMINE, GEPIA, LinkedOmics, and COEXPEDIA.

**Results:**

According to the results, the mRNA expression of SLC2A1 was significantly higher in breast cancer, while the expression levels of SLC2A2–4 were downregulated. The results also indicate that GLUT1 expression does not have significant influence on the overall survival of patients with breast cancer. The mRNA expression of *SLC2A1* and *RB1* is significantly correlated, which means that tissues with high *RB1* mRNA expression might have relatively higher mRNA expression of *SLC2A1*; however, further study analyzing their roles in the expression regulation pathways with human samples is needed to verify the hypothesis.

**Conclusions:**

The mRNA expression of *SLC2A1* was significantly higher in breast cancer. The overall survival of breast cancer patients wasn’t significantly correlated with GLUT1–4 expression. The mRNA expression of SLC2A1 and RB1 is significantly correlated according to the analysis conducted in LinkedOmics. It provides reference for future possible individualized treatment of TNBC using GLUT1 inhibitors, especially in patients with higher mRNA expression of *RB1*. Further study analyzing the roles of these two genes in the regulation pathways is needed.

**Supplementary Information:**

The online version contains supplementary material available at 10.1186/s12885-021-08763-y.

## Background

Glucose Transporters (GLUTs) proteins are encoded by the SLC2 genes and are members of the major facilitator superfamily of membrane transporters [1]. GLUTs are the main facilitators of glucose transport in mammalian cells [2]. Fourteen GLUT proteins are expressed in humans and they can be categorized into three classes based on sequence similarity: Class 1 (GLUTs 1–4, 14), Class 2 (GLUTs 5, 7, 9, and 11), and Class 3 (GLUTs 6, 8, 10, 12, and HMIT) [3]. Several studies have shown that GLUT1 expression is increased in a variety of malignant tumors [4–6]. This is probably because tumor cells show an enhanced level of glucose metabolism compared to normal tissues, and tumor cells have greater need for glucose, which results in a corresponding increase in the transport of glucose into the cells. In addition, it has been reported that GLUT1 overexpression is closely related to tumor progression and is related to the poor prognosis of a variety of malignant tumors [7–9].

Current treatment methods for triple-negative breast cancer (TNBC) patients are very limited [10], and the prognosis of TNBC is relatively poor [11]. Human glucose transporter 1 (hGLUT1) is overexpressed in breast cancer tissues. A series of GLUT1 inhibitors have been discovered [12–16], and these molecules have the potential to block glucose transport in breast cancer tissue and treat TNBC. However, recent research has found that not all types of TNBC cells are sensitive to GLUT1 inhibitor [17]. Different breast cancer cells showed diverse sensitivities to GLUT1 inhibitors, and the protein level of RB1 strongly correlated with the degree of sensitivity to GLUT1 inhibition in TNBC. It was established in a recently published TNBC related research that RB1-negative cells were insensitive to GLUT1 inhibition [17]. According to the research, the effect of GLUT1 inhibitors on the inhibition of TNBC cells depended largely on the RB1 expression level of the cancer tissue and cells. Based on existing research conclusions, we put forward a hypothesis that there may be a close correlation between the expression of GLUT family, especially the expression of GLUT1–4, and the expression of RB1.

To the best of our knowledge, there has been no study reporting the expression and prognosis analyses of GLUT1–4 (encoded by genes *SLC2A1-SLC2A4*) in breast cancer using data mining. In this study, we used public databases such as ONCOMINE, GEPIA, LinkedOmics, and COEXPEDIA. We systematically studied the effect of GLUT1–4 gene expression level on the prognosis of breast cancer, and explored the possible relationship between the expression of GLUT1–4 and RB1 in breast cancer tissues. The study provides a reference for future possible treatment of TNBC using GLUT1 inhibitors.

## Methods

In this study, public databases such as ONCOMINE, GEPIA, LinkedOmics, and COEXPEDIA were used to systematically study the co-expression of GLUT1–4, the influence of GLUT1–4 gene expression on the prognosis of breast cancer, and to explore the possible relationship between the expression of GLUT1–4 and RB1 in breast cancer tissues.

### ONCOMINE analysis

ONCOMINE gene expression array database (https://www.oncomine.org/) is an online cancer microarray database. In this study, it was used to analyze the transcription levels of *SLC2A1–4* genes in different cancers. The mRNA expression levels of *SLC2A1–4* were especially compared between clinical breast cancer samples and normal controls, using a Student’s t test to generate the *p*-value. The cutoff values of p and fold change were respectively defined as 1 × 10^− 4^ and 2. ONCOMINE was also used for gene co-expression analyses of the four GLUT family genes.

### GEPIA dataset

GEPIA (Gene Expression Profiling Interactive Analysis) is a newly developed interactive web server for analyzing the RNA sequencing expression data of 9736 tumors and 8587 normal samples from the TCGA and the GTEx projects, using a standard processing pipeline. GEPIA provides customizable functions such as tumor/normal differential expression analysis, profiling according to cancer types or pathological stages, patient survival analysis, similar gene detection, correlation analysis, and dimensionality reduction analysis [18]. In our study, GEPIA was used to analyze the mRNA levels of *SLC2A1–4* in breast cancer vs. normal tissues. Scatter diagrams, bar charts, and box plots were automatically generated according to the combined conditions put into the website. GEPIA was also used to conduct survival analyses and to correlation analyses between two genes.

### LinkedOmics dataset

LinkedOmics (http://www.linkedomics.org/login.php) is a publicly available portal that includes multi-omics data from all 32 TCGA cancer types. It also includes mass spectrometry-based proteomics data generated by the Clinical Proteomics Tumor Analysis Consortium for TCGA breast, colorectal, and ovarian tumors [19]. In this study, LinkedOmics was used to conduct OS analyses in relation to GLUT1–4 expression. It was also used in the correlation analyses among genes *SLC2A1–4* and *RB1*.

### COEXPEDIA

Massive amounts of array-based transcriptomics data have been deposited in several public depositories such as Gene Expression Omnibus (GEO) and ArrayExpress. COEXPEDIA is a database of context-associated co-expression networks inferred from an individual series of microarray samples for humans and mice of GEO. COEXPEDIA is a distinctive co-expression database by the following three aspects: 1) All co-expression links were evaluated for functional association by statistical assessment. 2) All co-expression links are associated with particular biomedical contexts. 3) All co-expression links have associated medical subject heading terms, which provide anatomical or disease context information [20]. In our study, COEXPEDIA was used to conduct correlation analyses among genes.

## Results

### Transcriptional levels of *SLC2A1*-*SLC2A4* (GLUT1–4) in patients with breast cancer

**Fig. 1:**
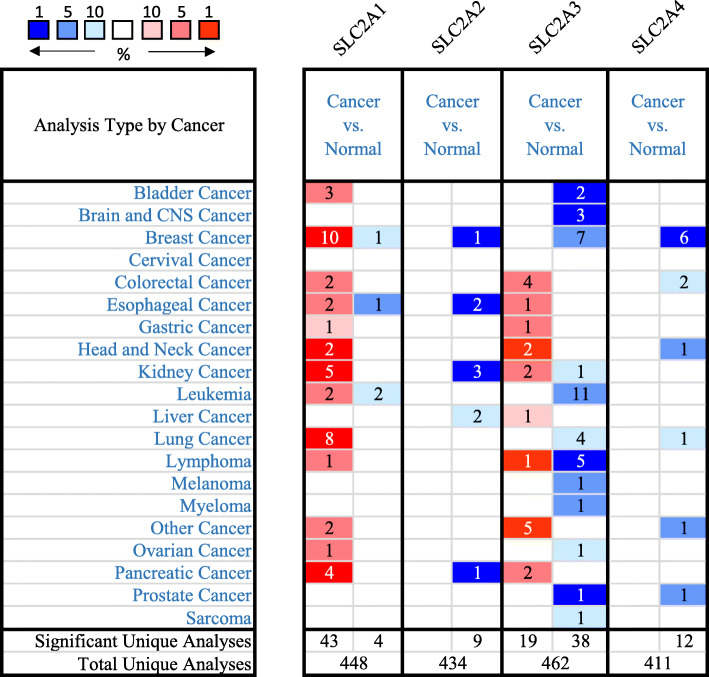
The Transcription Levels of GLUT1–4 in different types of cancers (ONCOMINE)

The mostly studied GLUTs in humans are GLUT1–4. The transcriptional levels of the corresponding genes *SLC2A1* through four in cancers are compared with those in normal samples by using ONCOMINE database. The disease summary of the transcriptional levels of *SLC2A1–4* is shown in Fig. 1. As is shown in the figure, 10 out of 53 analyses (4 out of 14 datasets) revealed SLC2A1 upregulation in breast cancer, while 1 out of 53 analyses (1 out of 14 datasets) displayed SLC2A1 downregulation. As is shown in Table 1, the expression levels of GLUT1 were significantly upregulated in patients with different subtypes of invasive and non-invasive breast cancer in four datasets. In the Zhao Breast dataset [21], *SLC2A1* was overexpressed in invasive ductal breast carcinoma and lobular breast carcinoma compared with that in the normal samples, with a fold change of 2.800 and 2.075 separately. In the TCGA Breast dataset [22], *SLC2A1* was overexpressed compared with that in the normal samples in intraductal cribriform breast adenocarcinoma (fold change = 2.172), in male breast carcinoma (fold change = 3.575), in invasive ductal breast carcinoma (fold change = 2.557) and in invasive breast carcinoma (fold change = 2.251). In the Richardson Breast 2 [23] dataset, SLC2A1 was also overexpressed in ductal breast carcinoma with a fold change of 2.340. The Curtis Breast dataset [24] indicated that compared to normal samples, SLC2A1 overexpression is also found in medullary breast carcinoma (fold change = 2.728), in mucinous breast carcinoma (fold change = 2.100) and in invasive breast carcinoma (fold change = 2.317). (Table 1) Conversely, the transcriptional levels of SLC2A2–4 were not significantly upregulated, but showed downregulation in breast cancer (Fig. 1).

**Table 1:** The Significant Changes of SLC2A1 Expression in Transcription Level between Different Types of Breast Cancer (ONCOMINE Database)

Gene ID	Types of Breast cancer versus Normal	Fold Change	*P* Value	t Test	References
SLC2A1	Invasive Ductal Breast Carcinoma versus Normal	2.800	1.03E-11	9.276	Zhao Breast
	Lobular Breast Carcinoma versus Normal	2.075	6.62E-6	5.631	Zhao Breast
	Intraductal Cribriform Breast Adenocarcinoma versus Normal	2.172	2.50E-09	11.263	TCGA Breast
	Male Breast Carcinoma vs. Normal	3.575	2.84E-5	11.010	TCGA Breast
	Invasive Ductal Breast Carcinoma vs. Normal	2.557	4.19E-27	13.974	TCGA Breast
	Invasive Breast Carcinoma vs. Normal	2.251	8.98E-15	8.629	TCGA Breast
	Ductal Breast Carcinoma vs. Normal	2.340	1.07E-6	6.053	Richardson Breast 2
	Medullary Breast Carcinoma vs. Normal	2.728	4.82E-10	8.059	Curtis Breast
	Mucinous Breast Carcinoma vs. Normal	2.100	6.44E-13	8.635	Curtis Breast
	Invasive Breast Carcinoma vs. Normal	2.317	1.49E-5	5.207	Curtis Breast

### Relationship between the mRNA levels of *SLC2A1–4* and the clinicopathological parameters of patients with breast cancer

**Fig. 2:**
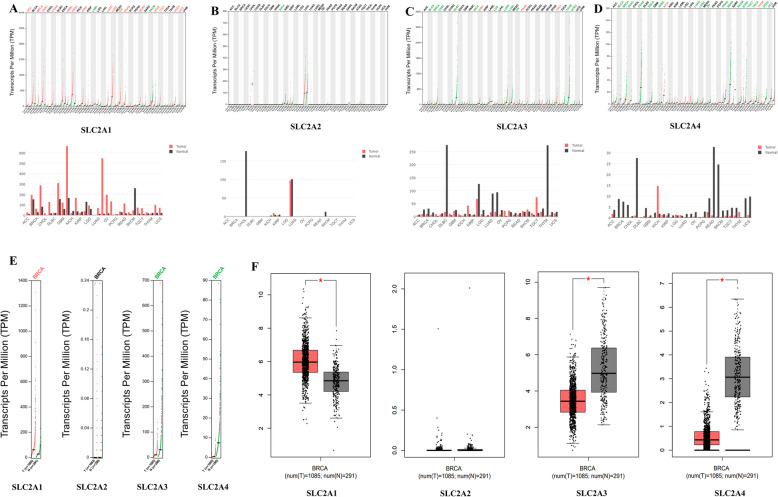
The expression of SLC2A1–4 in pan-cancer and breast cancer (GEPIA). (A)-(D): The expression of SLC2A1–4 (GLUT1–4) in pan-cancer (BRCA: Breast invasive carcinoma); (E)-(F): The expression of SLC2A1–4 in breast cancer (BRCA: Breast invasive carcinoma). Please see supplementary Table S1 for all the cancer type abbreviations in PEGIA

The GEPIA (Gene Expression Profiling Interactive Analysis) dataset was used to compare the mRNA expression of *SLC2A1–4* between breast cancer and normal tissue samples. Each of Fig. 2A-D consisted of 2 diagrams: the corresponding gene expression profile across all tumor samples and paired normal tissues (dot plot: with each dot representing expression of samples; and bar plot: with the height of bar representing the median expression of certain tumor type or pairing normal tissue). Figure 2E is the dot plot revealing the expression profile of SLC2A1–4 in breast invasive carcinoma, with each dot representing expression of samples; Fig. 2F is the bar plot displaying the expression profile of SLC2A1–4 in breast cancer, with the height of bar representing the median expression of certain tumor type or pairing normal tissue. The results showed that the expression level of *SLC2A1* were higher in breast invasive carcinoma than in pairing normal tissues, and the expression levels of *SLC2A3* and *SLC2A4* were significantly lower in breast invasive carcinoma than in pairing normal tissues (Fig. 2A-F).

### The prognostic values of SLC2A1–4 and RB1 in breast cancer

As mentioned in the Introduction, it has been suggested that RB1 expression in TNBC might be used as a biomarker for the inhibitory effect of GLUT1 inhibitors on breast cancer. Here, we used GEPIA and LinkedOmics databases to investigate the prognostic value of *SLC2A1–4* and *RB1* gene expression in breast cancer. We generated survival curves reflecting the relationship between the overall survival (OS) rate of the patients and the corresponding gene expression levels.

**Fig. 3:**
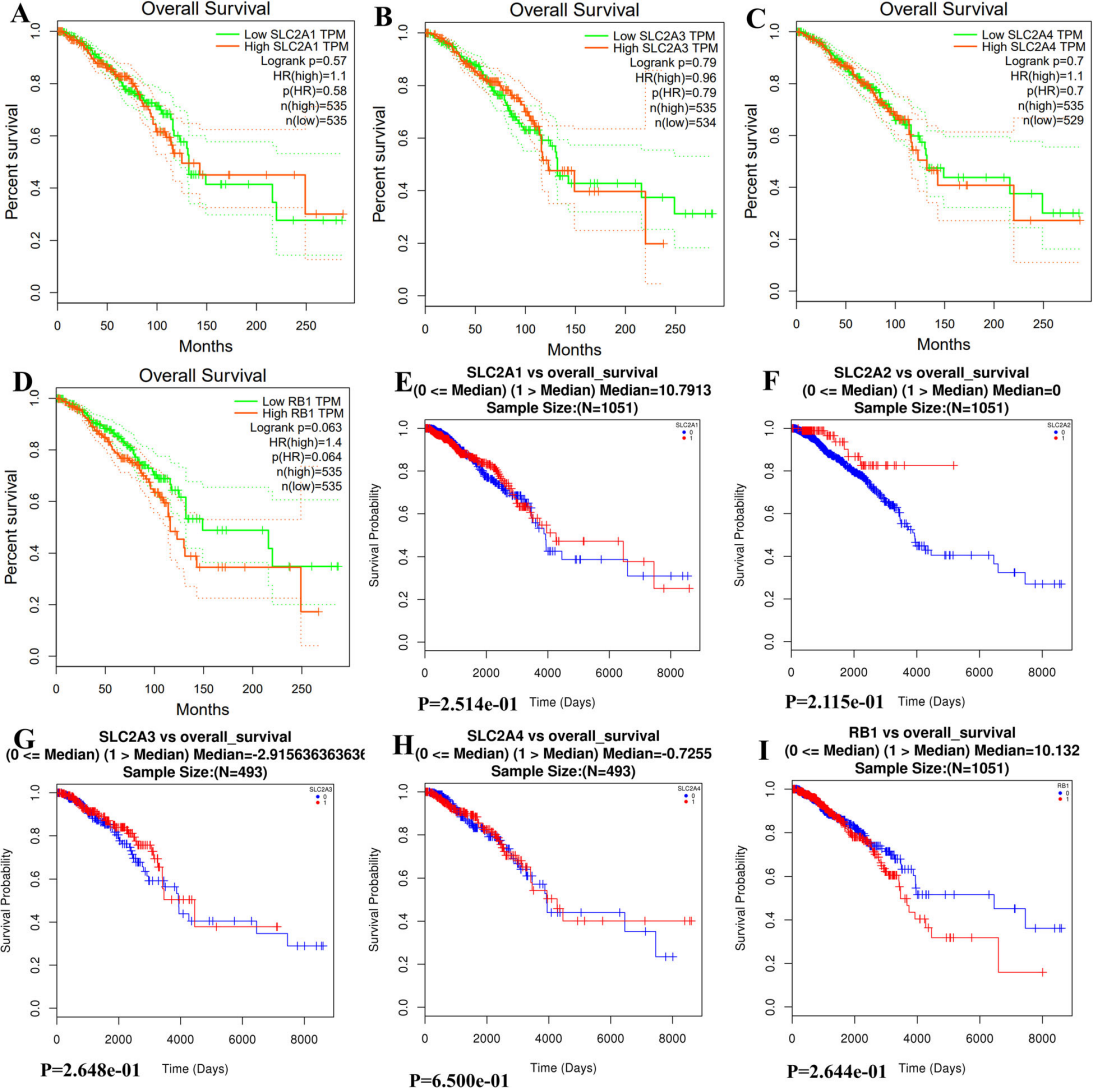
Survival Curves of gene expression levels of SLC2A1–4 and RB1. Survival Curves of gene expression levels of SLC2A1,3,4 and RB1(A-D) in breast cancer analyzed with GEPIA; Survival curves of gene expression levels of SLC2A1–4,RB1 (E-I) in breast cancer analyzed with LinkedOmics

Survival curves generated in GEPIA for *SLC2A1,3,4* and *RB1* are shown in Fig. 3A-D. The sample size was insufficient to generate a survival curve for *SLC2A2*. According to shape of the curves shown in the figure, decreased *RB1* might be associated with poor OS in breast cancer, but its *p* value showed no significance (*p* > 0.05). Survival curves generated in LinkOmics [19] for *SLC2A1–4*, *RB1* are shown in Fig. 3E-I. As we can see from the shape of the curves, decreased *RB1* might be associated with poor OS in breast cancer, but the *p* value showed no significance (*p* > 0.05). *SLC2A1–4* expression level does not have a significant influence on the OS in breast cancer.

### Co-expression gene analyses for *SLC2A1–4*

**Fig. 4:**
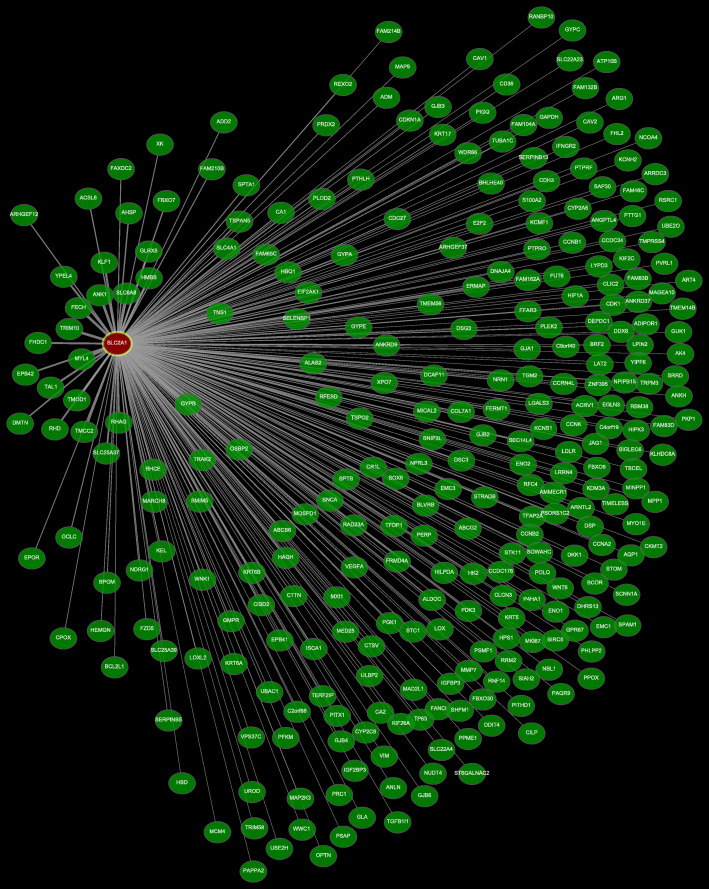
Co-expression network of SLC2A1 (Coexpedia) The smaller distance between the linked genes, the more probable that the gene pairs were co-expressed.

Genes co-expressed with *SLC2A1–4* were analyzed using the COEXPEDIA website. The co-expressed network of *SLC2A1* is shown in Fig. 4, and the co-expressed network figures of *SLC2A2–4* are in the Supplementary materials (Fig. S1, S2 and S3). The sum of log likelihood scores from all co-expression links (LLS score) are listed in supplementary Table S2. The smaller distance between the linked genes in the figures, the higher LLS score they had in the table, the more probable that the corresponding gene pairs were co-expressed. According to the results, the top six genes found to be co-expressed with *SLC2A1* were *MYL4, SLC6A8, ANK1, TRIM10, FECH,* and *GYPB*, with the sum of log likelihood scores from all co-expression links (LLS score) of 29.435, 28.116, 25.847, 25.183, 24.849, and 23.899. The top six genes shown to be co-expressed with *SLC2A2* were *KNG1, HRG, SERPINC1, MAT1A, ALDOB,* and *CFHR2*, with the sum of edges’ LLS score of 16.558, 15.079, 14.500, 14.474, 13.897, and 13.729. The top six genes analyzed to be co-expressed with *SLC2A3* were *MAFF, MCL1, FOSL2, PLAUR, NR4A2,* and *BHLHE40*, with scores of 36.855, 33.949, 30.075, 29.411, 27.156, and 27.073. Only five genes were shown to be co-expressed with *SLC2A4*, including *PFKFB1, ADAM23, AQP5, SH2D3C,* and *TTYH2*, with scores of 1.862, 1.803, 1.303, 1.261, and 1.137 respectively.

**Fig. 5:**
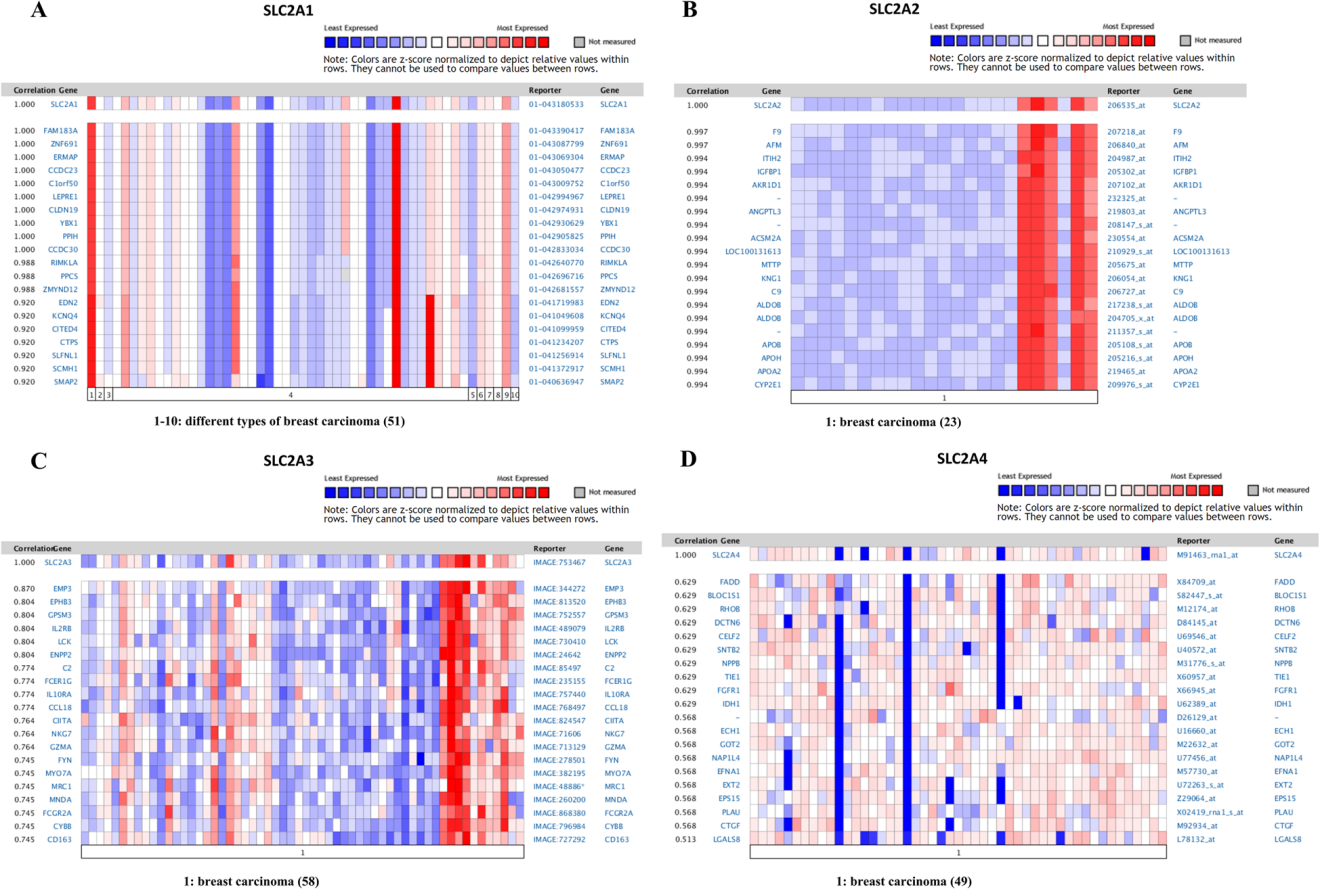
Co-expressed genes of SLC2A1–4, analyzed by ONCOMINE. The correlation scores on the left side of the figures represent the level of co-expression between SLC2A1 or SLC2A2–4 with other genes

Subsequently, we also conducted co-expression gene analyses on ONCOMINE; the co-expression color maps are shown in Fig. 5(A-D). Genes co-expressed with *SLC2A1* were analyzed in Haverty Breast [25], and the results showed that *SLC2A1* is co-expressed with *FAM183A, ZNF691, ERMAP, CCDC23, C1orf50, LEPRE1, CLDN19, YBX1, PPIH, CCDC30, RIMKLA*, etc. (Fig. 5A). Genes co-expressed with *SLC2A2* were analyzed in Landemaine Breast [26]; the result of which showed that SLC2A2 is co-expressed with *F9, AFM, ITIH2, IGFBP1, AKR1D1, ANGPTL3, ACSM2A, LOC100131613, MTTP, KNG1, C9, ALDOB*, etc. (Fig. 5B). Gene co-expression analyses for *SLC2A3* were conducted with Gruvberger Breast [27], and the results showed that *SLC2A3* is co-expressed with *EMP3, EPHB3, GPSM3, IL2RB, LCK, ENPP2, C2, FCER1G, IL10RA, CCL18, CIITA*, etc. (Fig. 5C). Gene co-expression analyses for SLC2A4 were conducted with West Breast [28], and the results showed that *SLC2A4* is co-expressed with *FADD, BLOC151, RHOB, DCTN6, CELF2, SNTB2, NPPB, TIE1, FGFR1, IDH1, ECH1*, etc. (Fig. 5D).

### Correlation analyses among *SLC2A1–4* and *RB1*

**Fig. 6:**
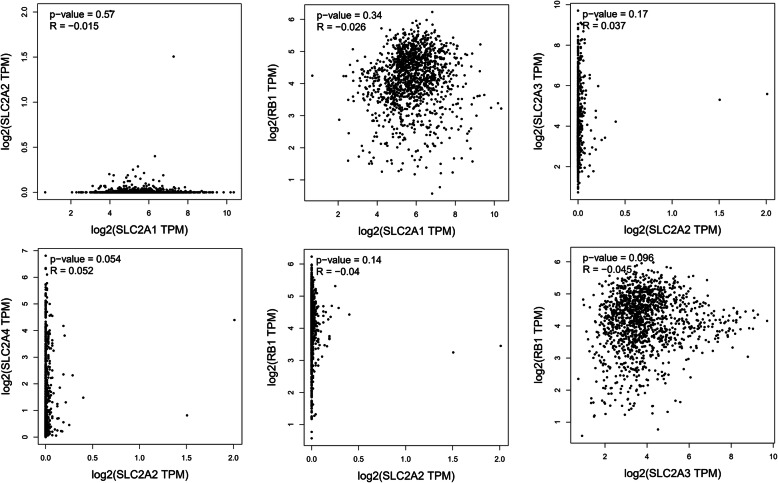
Positive correlation results between SLC2A1–4 and RB1 in breast cancer (conducted using GEPIA: a-d, and conducted using LinkedOmics: e-j). a: SLC2A1-SLC2A3, b: SLC2A1-SLC2A4, c: SLC2A3-SLC2A4, d: SLC2A4-RB1; e: SLC2A1-SLC2A3, f: SLC2A1-SLC2A4, g: SLC2A-RB1, h: SLC2A2-SLC2A4, i: SLC2A3-SLC2A4, j: SLC2A3-RB1

Finally, we analyzed the possible association among *SLC2A1–4* and *RB1*, using LinkedOmics database and PEGIA. All the *P* values are shown in Table 2, *P* values < 0.05 were seen as results indicating significant correlation between genes. The positive results analyzed in PEGIA and LinkedOmics are shown in Fig. 6. The negative results are shown in Supplementary materials (Fig. S4 and S5). As is shown in Fig. 6, the positively associated gene pairs included: *SLC2A1-SLC2A3*, *SLC2A1-SLC2A4*, *SLC2A3-SLC2A4,* and *SLC2A4-RB1* in PEGIA analyses (Fig. 6 a-d); and *SLC2A1-SLC2A3*, *SLC2A1-SLC2A4*, *SLC2A1-RB1*, *SLC2A2-SLC2A4*, *SLC2A3-SLC2A4,* and *SLC2A3-RB1* in LinkedOmics analyses (Fig. 6e-j). As is shown in Table 2, the RNA expression of some gene pairs was significantly correlated in both PEGIA and LinkedOmics analyses. These gene pairs are: *SLC2A1-SLC2A3*, *SLC2A1-SLC2A4,* and *SLC2A3-SLC2A4*. This indicates that GLUT1 is significantly correlated with GLUT3 and GLUT4, and GLUT3 is also significantly correlated with GLUT4. Four other gene pairs had positive results, which only showed significant positive results in one of the database analyses (either in PEGIA or in LinkedOmics). The correlation between these gene pairs might need further investigation and confirmation. These gene pairs included: *SLC2A1-RB1*, *SLC2A3-RB1*, *SLC2A4-RB1,* and *SLC2A2-SLC2A4*.

**Table 2:** Results (*p-*values) of gene pairwise correlation analyses in GEPIA (data in the top right part of the table) and LinkedOmics (data in the bottom left part of the table). Data representing significant correlation between genes are shown in bold (*: *p* < 0.05, **:*p* < 0.01)

*p*-value	SLC2A1	SLC2A2	SLC2A3	SLC2A4	RB1
SLC2A1		0.57	0.018*	**1.0 × 10**^−11^**	0.34
SLC2A2	0.31		0.17	0.054	0.14
SLC2A3	**1.0 × 10**^−4^**	0.25		**0.90 × 10**^−12^**	0.096
SLC2A4	**3.5 × 10**^−3^**	0.011*	**3.9 × 10**^−12^**		**1.0 × 10**^−4^**
RB1	**2.4 × 10**^−13^**	0.26	**1.2 × 10**^−4^**	0.24

## Discussion

The expression of GLUT1–4 has been reported in many cancers [29]. The present study is the first to explore the relationship of mRNA expression between *SLC2A1–4* and *RB1*, and to study the prognostic values of GLUT1–4 in breast cancer using data mining methods. We hope that our findings can contribute to available knowledge, report the expression of GLUT1–4 in breast cancer, and more importantly, provide a reference for the potential individualized metabolic inhibition therapy of TNBC using hGLUT1 inhibitors.

In our study, the mRNA expression of *SLC2A1* was significantly higher in breast cancer, while the expression levels of SLC2A2–4 were downregulated. The result is in accordance with previously published literature, which state that GLUT1 is crucial for uptake of glucose by breast cancer cells, and is also the main glucose transporter in breast cancer cell lines [30]. Although it has also been reported that a strong correlation between GLUT1 gene expression and breast cancers of higher grade and proliferative index and lower degree of differentiation [31] and higher malignant potential, invasiveness, and consequently poorer prognosis [32] exists, the *p*-values in our prognosis analyses were all larger than 0.05. The OS of patients with breast cancer was not significantly correlated with GLUT1–4 expression. With 20 years’ survival data of more than 1000 subjects included in the analyses, we think of the results to be quite convincing. It is considered that the relationship between the expression of GLUT1 and the OS of patients with breast cancer is not clear. Further evidence is required to determine whether GLUT1 can be used as a prognostic biomarker for breast cancer.

Moreover, in terms of the correlation between GLUT1 and RB1 expression, the analysis conducted in LinkedOmics had a positive result for this gene pair, with a sample size of 1093 and *p-*value of 2.429 × 10^− 13^. The result indicates that the mRNA expression of SLC2A1 and RB1 is significantly correlated. Further study analyzing their roles in the expression regulation pathways is required.

## Conclusions

The mRNA expression of SLC2A1 was significantly higher in breast cancer. The overall survival of breast cancer patients wasn’t significantly correlated with GLUT1–4 expression. The mRNA expression of SLC2A1 and RB1 is significantly correlated according to the analysis conducted in LinkedOmics. It provides reference for future possible individualized treatment of TNBC using GLUT1 inhibitors, especially in patients with higher mRNA expression of *RB1*. Further study analyzing the roles of these two genes in the regulation pathways is needed.

## Supplementary Information

**Additional file 1: Figure S1**. Co-expression network of SLC2A2 (Coexpedia)

**Additional file 2: Figure S2**. Co-expression network of SLC2A3 (Coexpedia)

**Additional file 3: Figure S3**. Co-expression network of SLC2A4 (Coexpedia)

**Additional file 4: Figure S4**. Negative results of the correlation analyses in PEGIA

**Additional file 5: Figure S5**. Negative results of the correlation analyses in LinkedOmics

**Additional file 6: Table S1**. List of Abbreviations for all cancer types in PEGIA database

**Additional file 7: Table S2**. LLS scores of gene co-expression analyses for SLC2A1–4 in COEXPEDIA

## Data Availability

The datasets analysed during the current study are available in ONCOMINE, GEPIA, LinkedOmics, and COEXPEDIA. [https://www.oncomine.org/;http://gepia.cancer-pku.cn/index.html; http://www.linkedomics.org/login.php; https://www.coexpedia.org/]. All data and outcomes generated during this study are included in this published article and its supplementary information files.

## Abbreviations

TNBC: Triple negative breast cancer
GLUTs: Glucose transporters
GLUT1: Glucose transporter 1
RB1: Retinoblastoma gene 1
GEPIA: Gene Expression Profiling Interactive Analysis
TCGA: The Cancer Genome Atlas
