# Supplementary figures and images for: Co-expression and prognosis analyses of GLUT1–4 and RB1 in breast cancer
Source: BMC Cancer. 2021 Sep 15;21:1026. doi: 10.1186/s12885-021-08763-y (PMC8442321; doi:10.1186/s12885-021-08763-y)

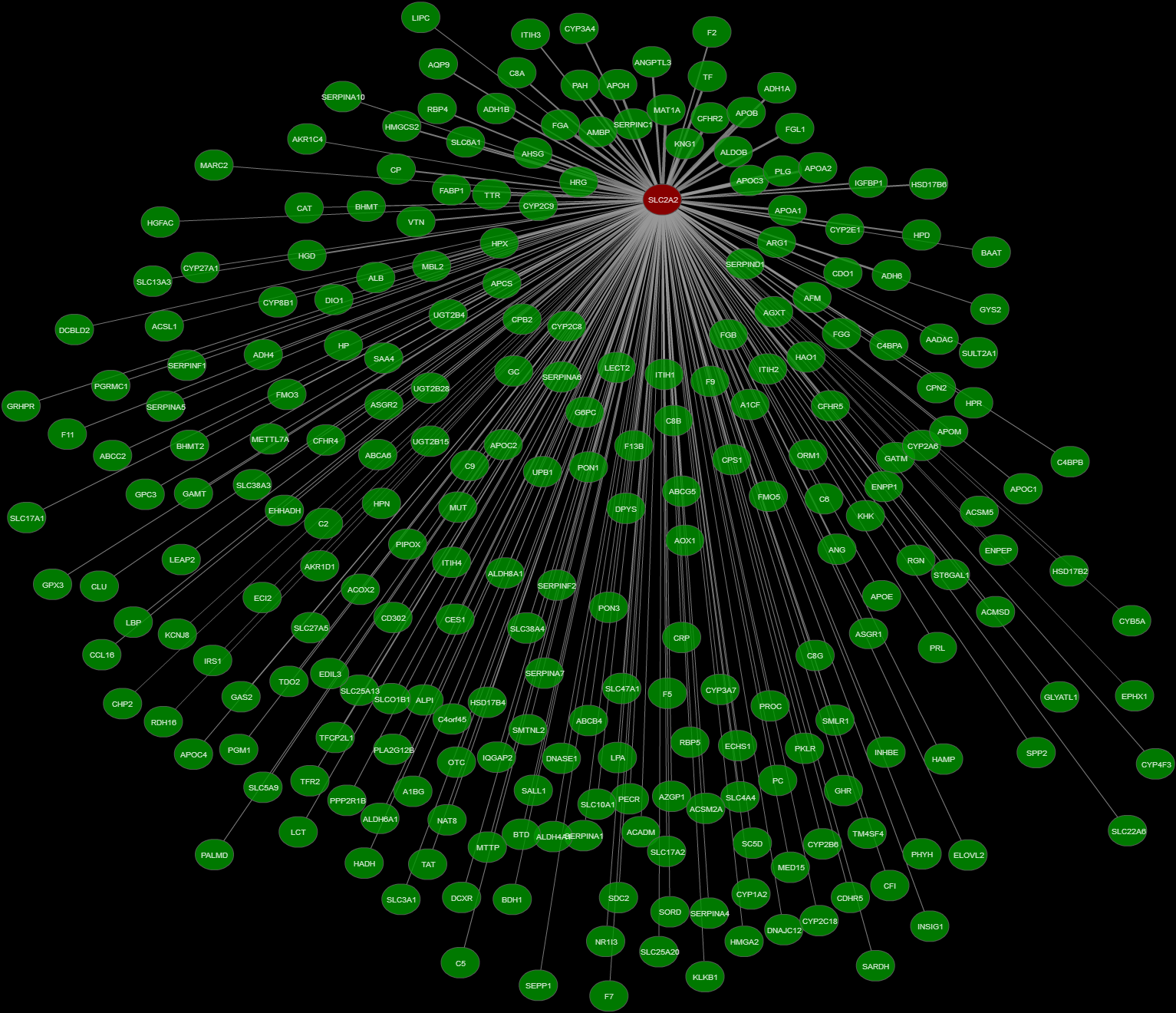

Supplement: Supplementary file 1 — Additional file 1: Figure S1. Co-expression network of SLC2A2 (Coexpedia) [file 12885_2021_8763_MOESM1_ESM.png]

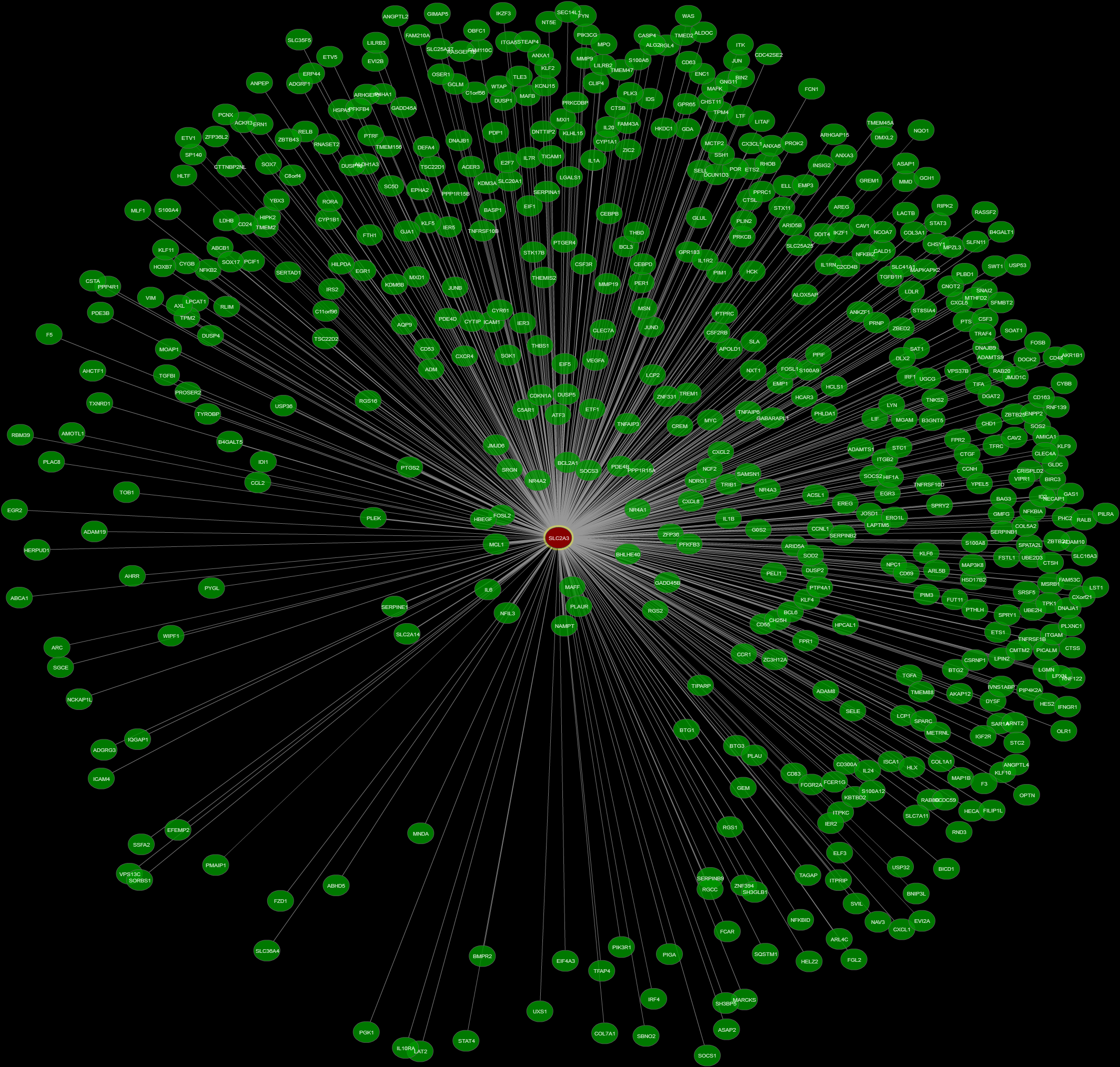

Supplement: Supplementary file 2 — Additional file 2: Figure S2. Co-expression network of SLC2A3 (Coexpedia) [file 12885_2021_8763_MOESM2_ESM.png]

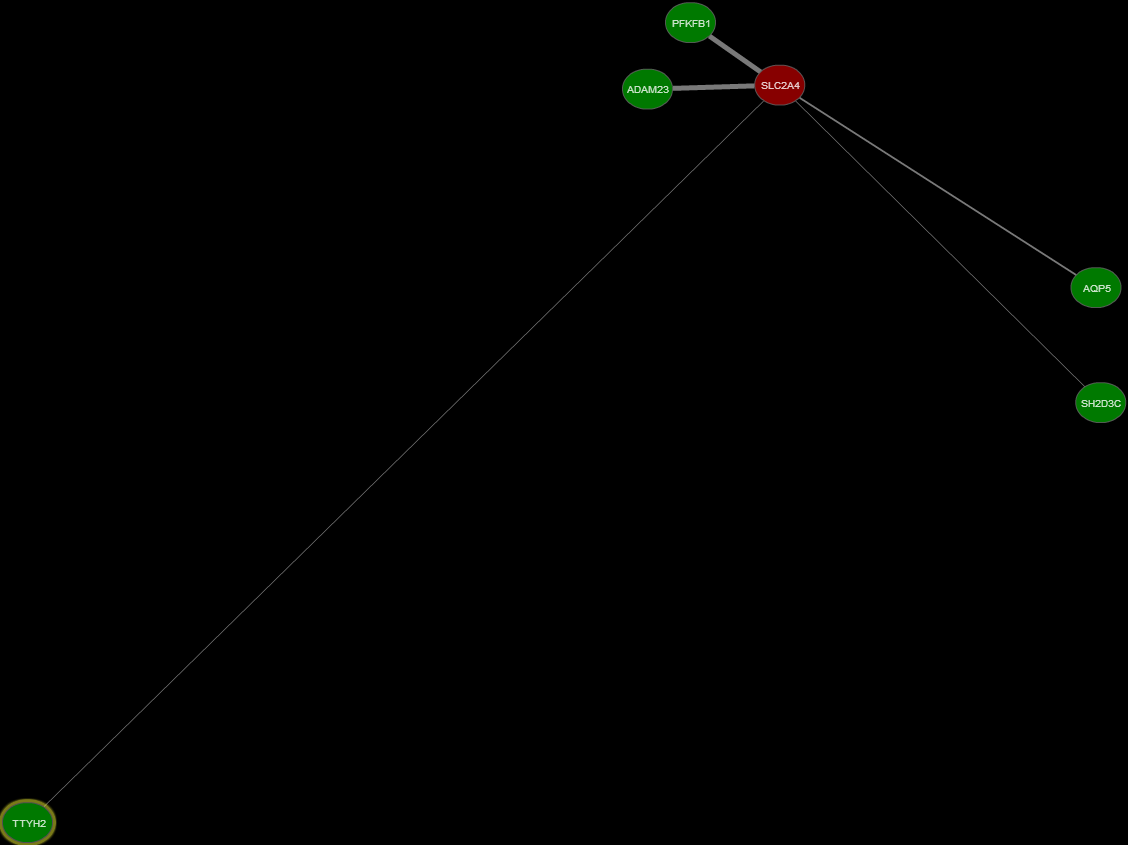

Supplement: Supplementary file 3 — Additional file 3: Figure S3. Co-expression network of SLC2A4 (Coexpedia) [file 12885_2021_8763_MOESM3_ESM.png]

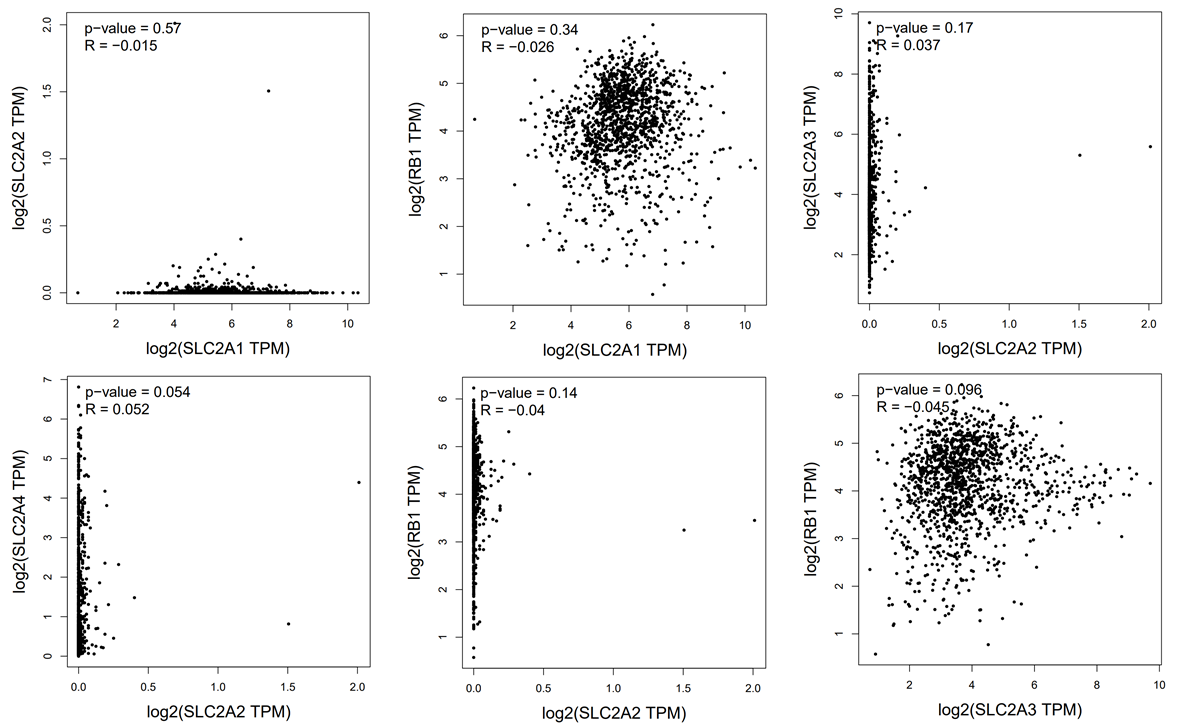

Supplement: Supplementary file 4 — Additional file 4: Figure S4. Negative results of the correlation analyses in PEGIA [file 12885_2021_8763_MOESM4_ESM.tif]

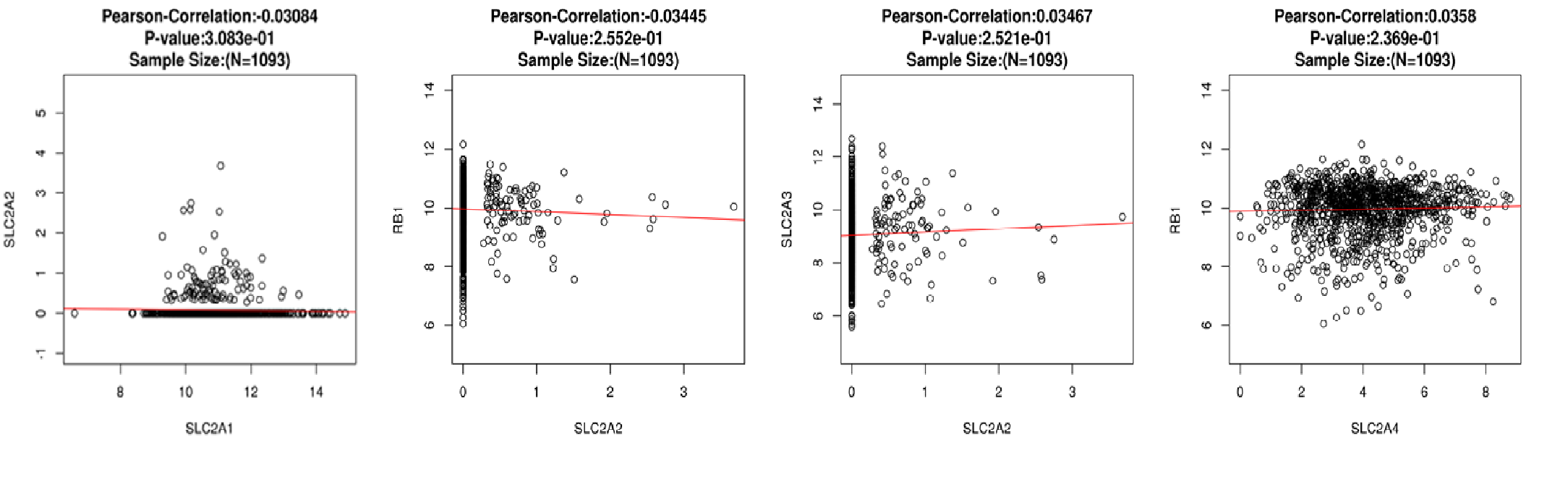

Supplement: Supplementary file 5 — Additional file 5: Figure S5. Negative results of the correlation analyses in LinkedOmics [file 12885_2021_8763_MOESM5_ESM.tif]
