# Supplementary material for: Co-expression and prognosis analyses of GLUT1–4 and RB1 in breast cancer
Source: BMC Cancer. 2021 Sep 15;21:1026. doi: 10.1186/s12885-021-08763-y (PMC8442321; doi:10.1186/s12885-021-08763-y)
Supplement: Supplementary file 7 — Additional file 7: Table S2. LLS scores of gene co-expression analyses for SLC2A1–4 in COEXPEDIA [file 12885_2021_8763_MOESM7_ESM.docx]

Table S2. LLS scores of gene co-expression analyses for SLC2A1-4 in COEXPEDIA.

| SLC2A1 co-expressed genes | LLS score |
| --- | --- |
| MYL4 | 29.435 |
| SLC6A8 | 28.116 |
| ANK1 | 25.847 |
| TRIM10 | 25.183 |
| FECH | 24.849 |
| GYPB | 23.899 |
| SLC2A2 co-expressed genes | LLS score |
| KNG1 | 16.558 |
| HRG | 15.079 |
| SERPINC1 | 14.500 |
| MAT1A | 14.474 |
| ALDOB | 13.897 |
| CFHR2 | 13.729 |
| SLC2A3 co-expressed genes | LLS score |
| MAFF | 36.855 |
| MCL1 | 33.949 |
| FOSL2 | 30.075 |
| PLAUR | 29.411 |
| NR4A2 | 27.156 |
| BHLHE40 | 27.073 |
| SLC2A4 co-expressed genes | LLS score |
| PFKFB1 | 1.862 |
| ADAM23 | 1.803 |
| AQP5 | 1.303 |
| SH2D3C | 1.261 |
| TTYH2 | 1.137 |
